# Emergence of a cholecystokinin/sulfakinin signalling system in Lophotrochozoa

**DOI:** 10.1038/s41598-018-34700-4

**Published:** 2018-11-06

**Authors:** Julie Schwartz, Marie-Pierre Dubos, Jérémy Pasquier, Céline Zatylny-Gaudin, Pascal Favrel

**Affiliations:** Normandie Université, UNICAEN, Sorbonne Universités, MNHN, UPMC, UA, CNRS 7208, IRD 207, Biologie des Organismes et Ecosystèmes Aquatiques (BOREA), CS14032, 14032 Caen, Cedex 5 France

## Abstract

Chordate gastrin/cholecystokinin (G/CCK) and ecdysozoan sulfakinin (SK) signalling systems represent divergent evolutionary scenarios of a common ancestral signalling system. The present article investigates for the first time the evolution of the CCK/SK signalling system in a member of the Lophotrochozoa, the second clade of protostome animals. We identified two G protein-coupled receptors (GPCR) in the oyster *Crassostrea gigas* (Mollusca), phylogenetically related to chordate CCK receptors (CCKR) and to ecdysozoan sulfakinin receptors (SKR). These receptors, Cragi-CCKR1 and Cragi-CCKR2, were characterised functionally using a cell-based assay. We identified di- and mono-sulphated forms of oyster Cragi-CCK1 (pEGAWDY(SO_3_H)DY(SO_3_H)GLGGGRF-NH_2_) as the potent endogenous agonists for these receptors. The Cragi-CCK genes were expressed in the visceral ganglia of the nervous system. The Cragi-CCKR1 gene was expressed in a variety of tissues, while Cragi-CCKR2 gene expression was more restricted to nervous tissues. An *in vitro* bioassay revealed that different forms of Cragi-CCK1 decreased the frequency of the spontaneous contractions of oyster hindgut. Expression analyses in oysters with contrasted nutritional statuses or in the course of their reproductive cycle highlighted the plausible role of Cragi-CCK signalling in the regulation of feeding and its possible involvement in the coordination of nutrition and energy storage in the gonad. This study confirms the early origin of the CCK/SK signalling system from the common bilaterian ancestor and delivers new insights into its structural and functional evolution in the lophotrochozoan lineage.

## Introduction

Neuropeptides include a diverse group of small neuron-signalling peptides that govern most animal biological functions and play a crucial role in the elaboration of adapted physiological and behavioural responses to environmental constraints. They activate specific receptors, which predominantly belong to the G protein-coupled receptor (GPCR) family. Global analyses including neuropeptide precursors and GPCR sequences from a variety of animal phyla have highlighted a long-term co-evolution of metazoan receptor-ligand families^1,2^. Until recently, knowledge on neuropeptide signalling systems was chiefly limited to well-studied vertebrate species and to ecdysozoan model species such as *Drosophila melanogaster* and *Caenorhabditis elegans*. Despite initial studies^3,4^, neuropeptide signalling has so far remained largely elusive in Lophotrochozoa, the protostomian sister clade of Ecdysozoa. Lophotrochozoa represent one of the most diverse and evolutionarily highly successful bilaterian lineages^5^. The neuropeptide repertoire of several lophotrochozoan species has now been established^6–9^, and large-scale genomic and transcriptomic resources are available^10,11^. This new situation offers the opportunity to investigate neuropeptide receptor coupling in a lophotrochozoan species and thus gain insight into the evolution of neuropeptide signalling in Bilateria. Accordingly, new signalling systems have recently been discovered in the annelid *Platynereis dumerilii*^12,13^ and in the mollusc *Crassostrea. gigas*^14–16^. The present study investigates the evolution of the gastrin/cholecystokinin (G/CCK)/sulfakinin (SK) signalling system in Lophotrochozoa, using the mollusc *C. gigas* as a representative species.

In chordates, the G/CCK family includes structurally conserved, frequently sulphated regulatory peptides^17^. Similarly to gut hormones, gastrin stimulates gastric acid secretion from the parietal cells of the stomach and plays a central role in gastric mucosa growth^18,19^, whereas CCK stimulates pancreatic enzyme secretion, induces the contraction and thus the emptying of the gallbladder, controls gut motility^20^, and stimulates satiety^21^. Besides these canonical functions in the regulation of digestive processes, CCK peptides now emerge as ubiquitous messengers due to the widespread expression of their encoding gene in a number of organs, including the brain where they are major neurotransmitters^22^ (for a review). The first protostomian member of the G/CCK family was biochemically characterised from head extracts of the cockroach (*Leucophea maderae*)^23^. This peptide was named leucosulfakinin (SK), and it exhibits around 55% of sequence identity with chordate G/CCKs. Moreover, the presence of a conserved sulphated tyrosyl residue further emphasises the resemblance with chordate G/CCKs. SKs have been identified in a number of insect species^24,25^ and crustaceans^26^. This family also includes the related nematode neuropeptide NLP12^27^. Like their vertebrate counterparts, they display potent myotropic activity in several tissues, especially the gut^28^ and the heart^29^. Similarly, they can regulate the release of digestive enzymes^30,31^ and play a role as satiety regulators^28,31–33^. In addition to their structural homology and their convergent biological activities with G/CCKs, SKs specifically activate receptors (SKRs) that share a common ancestral gene with CCK receptors (CCKRs)^34^.

Apart from initial studies reporting the occurrence of CCK-like immunoreactivity in the central nervous systems of *Aplysia californica*^35^ and *Nereis diversicolor*^36^, or revealing that insect SKs elicit the release of α-amylase from the gut of the scallop *Pecten maximus*^37^, the CCK/SK signalling system has remained unexplored in Lophotrochozoa.

The present study investigates a CCK/SK signalling system in Lophtrochozoa for the first time.

## Results

### Molecular characterization Cragi-CCKRs

**Figure 1:**
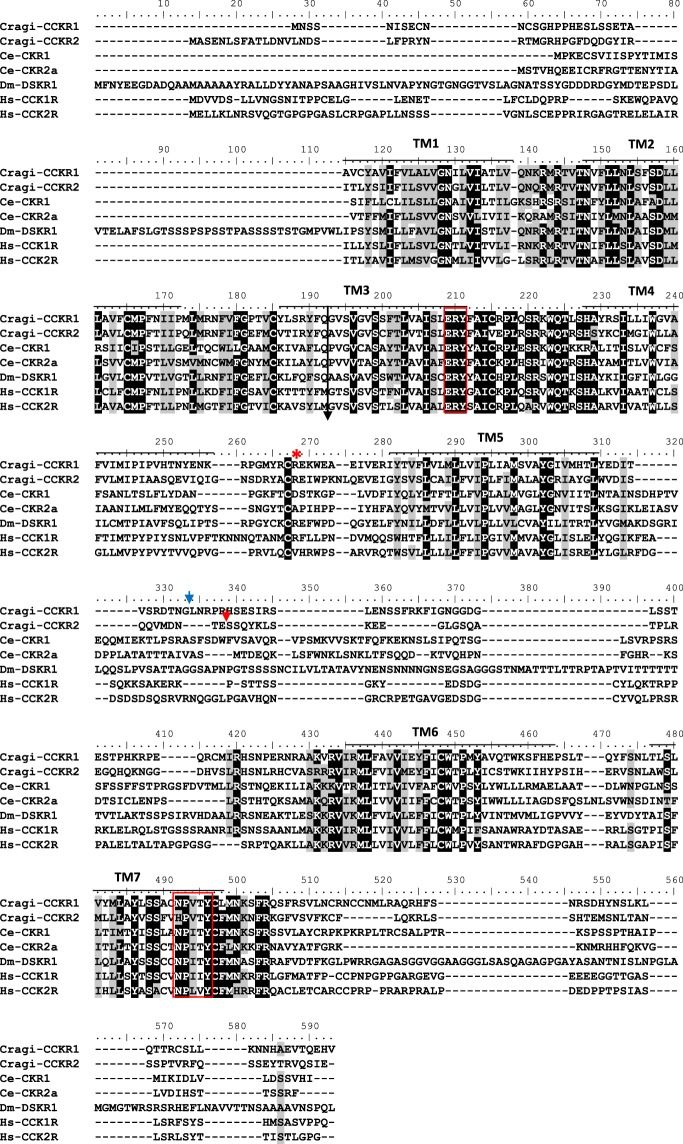
Alignment of the amino acid sequence of Cragi-CCKR1 (MF787221), Cragi-CCKR2 (MF787222) with CCKR family members from *Caenorhabditis elegans* (Ce-CKR1: NP_491918.3; Ce-CKR2a: ACA81683.1), *Drosophila melanogaster* (Dm-DSKR1: NP_001097021.1), *Homo sapiens* (Hs-CCKR1: NP_000721.1; Hs-CCKR2: NP_795344.1) using CLUSTALW^65^. Bars indicate the seven putative TM domains. Identical amino acid residues are highlighted in dark grey and similar residues in light grey. Black arrow indicates the position of a conserved intron, blue and red arrows indicate the respective position of Cragi-CCKR1 and Cragi-CCKR2 specific introns. Red boxes indicate the conserved “ERY” motif for receptor activation and “NPXXY” motif for Gαq and STAT –dependent signalling pathways. Red asterisk, the Arginine residue important for interaction with the sulphated tyrosine residue of CCK.

**Figure 2:**
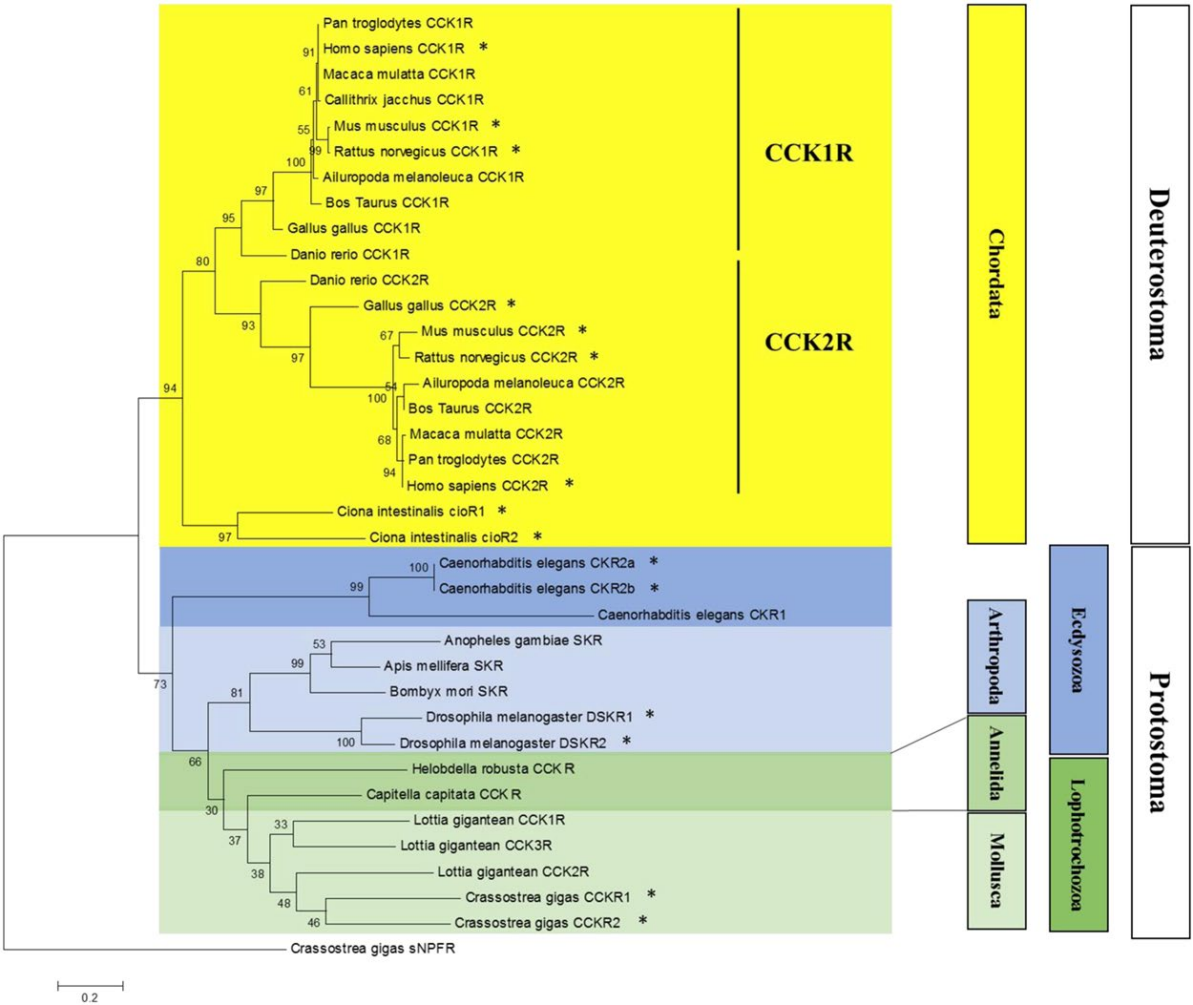
Phylogenetic representation of the relationship between Cragi-CCKRs and other CCKR family members. Phylogenetic and molecular evolutionary analyses were conducted using MEGA version 6^61^ based on the maximum likelihood method. The accession members of the sequences used to construct the tree are listed in Supplementary Table 2. The Cg-sNPFR^14^ was chosen as outgroup. * indicates functionally characterized receptors.

Two sequences displaying homologies with vertebrate and ecdysozoan CCK receptors were retrieved from GigaTON, an oyster comprehensive transcriptomic database^11^. These sequences named Cragi-CCKR1 and Cragi-CCKR2 share 43,5% of amino acid sequence identity. Cragi-CCKR1 and Cragi-CCKR2 also display around 31.5%-26.7% sequence identity with human CCKRs. Surprisingly, Cragi-CCKRs show slightly less identity with *C. elegans* CK-Rs (24.7-19.6%) and *Drosophila* DSKR1 (29.5–27.9%) (Fig. 1). In addition to the seven transmembrane domains characteristic of GPCRs, Cragi-CCKRs also hold the E/DRY and NPXXY (HPXXY for Cragi-CCKR2) motifs typical of family I GPCRs and critical for respectively receptor activation and Gαq dependent signalling pathways as well as STAT activation^38^. A phylogenetic analysis clearly revealed that Cragi-CCKR1 and Cragi-CCKR2 cluster with receptors from annelids and molluscs but as a separate branch from the insect SKRs. All chordate CCKRs form a distinct clade but with two branch clusters separating type 1 and type 2 receptors. *C. elegans* CKRs appeared more distant and emerged as a detached clade (Fig. 2). Alignment of the Cragi-CCKR cDNAs with the *C. gigas* genome sequence (http://www.oysterdb.com)^10^ identified Cragi-CCKR1 gene (scaffold1301, CGI_10027768) and Cragi-CCKR2 gene (scaffold962, CGI_10027668) and revealing the existence of an intron at a conserved position across vertebrate CCKR and insect SKR genes (Fig. 1)^1^.

### Molecular characterization of Cragi-CCKs

**Figure 3:**
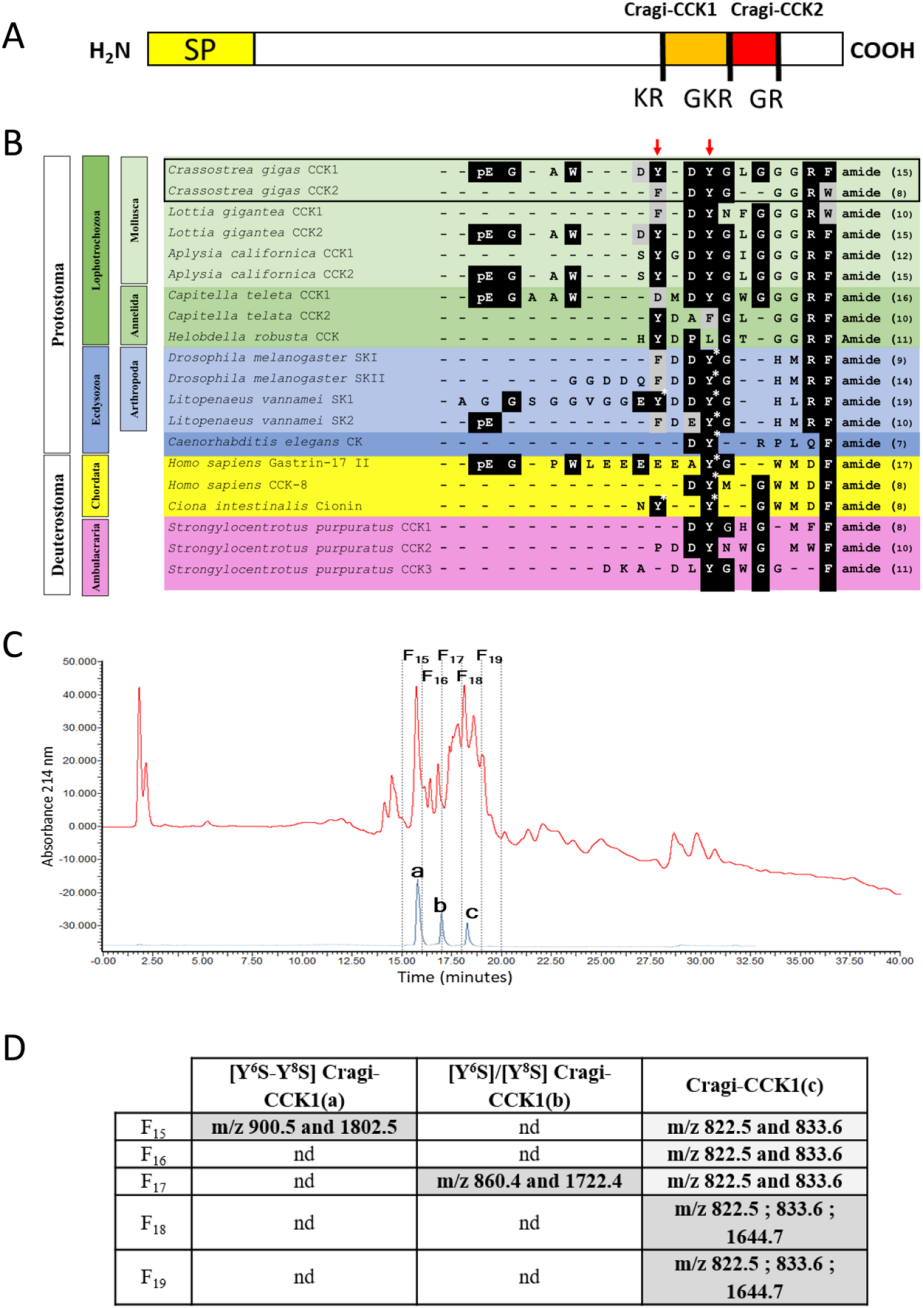
(**A**) Schematic representation of Cragi-CCK precursor^7^ (SP: Signal peptide). (**B**) Sequence alignment of Cragi-CCK peptides with Deuterostome G/CCK family members^44^, ecdysozoan sulfakinins (SK)/CK^27^ and lophotrochozoan CCK/SK^39,42^. The sulphated tyrosine residues (Y*) are labelled with an asterisk. Red-colored arrows indicate putative sulphated tyrosine residues in Cragi-CCKs or in putative CCK/SK peptides from other species. Black-colored and grey-colored shadings indicate respectively identical or similar amino acids (**C**) HPLC separation: In red, chromatogram of the second purification step of visceral ganglia extract. In blue, chromatogram of synthetic CCKs. a, [Y^6^S-Y^8^S] Cragi-CCK1; b, [Y^6^S]/[Y^8^S] Cragi-CCK1; c, Cragi-CCK1. (**D**) Detection with SIM mode of singly charged and doubled charged *ions* of different forms of Cragi-CCK1s in the different chromatographic fractions. nd: not detected.

Within the *C. gigas* neuropeptide repertoire^7^, two potential CCK-like peptides, Cragi-CCK1 (pEGAWDYDYGLGGGRFamide) and Cragi-CCK2 (pEFDYGGGRWamide)^39^, encoded by a single precursor (Fig. 3A) were detected from visceral ganglia (VG) extracts by mass spectrometry^7^. Alignment of Cragi-CCKs with CCK and SK peptides from other species shows that Cragi-CCK1 displays the C-terminal RFamide sequence feature common to insect SKs and distinct from the DFamide motif found in all chordate CCKs (Fig. 3B). Instead, Cragi-CCK2 comprises a C-terminal RWamide motif. Membership of *C. gigas* peptides to the CCK/SK family is also suggested by the conserved position of a key tyrosine residue which sulphated forms are often critical to elicit biological activity. In addition to the conserved tyrosine (Y^8^, from the N-terminus), Cragi-CCK1 shows an extra tyrosine residue (Y^6^) at a position corresponding to a second sulphated tyrosine present in the white shrimp *Litopenaeus vannamei* SK1 and the protochordate *Ciona intestinalis* peptide Cionin^26,40^. Singularly, Cragi-CCK1 also displays a N-terminal sequence similarity to that of human gastrin-17. Using liquid chromatography (RP-HPLC) and mass spectrometry analysis, the occurrence of the non-sulphated form and of the mono-sulphated and disulphated forms of CCK1 was unambiguously confirmed in oyster visceral ganglia (Fig. 3C,D, Supplementary Fig. 1).

### Cragi-CCK1 forms specifically activate Cragi-CCKRs

**Figure 4:**
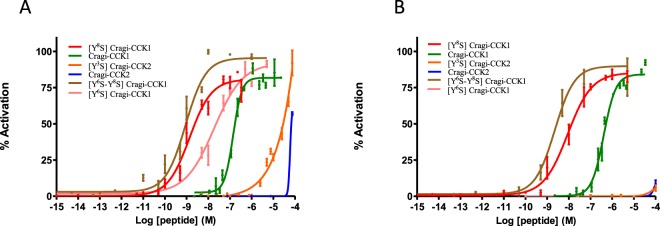
Dose-dependent activity of Cragi-CCK peptides on Cragi-CCKR1 (**A**) and on Cragi-CCKR2 (**B**) expressed in HEK293T cells. Data are shown as relative (%) to the highest value (100% activation) for a given peptide and represent the mean of an experiment (n = 4) performed in triplicate. Vertical bars represent the standard error of the mean (SEM).

Transiently transfected HEK293T cells expressing Cragi-CCKR1 or Cragi-CCKR2 with or without the promiscuous Gα16 protein were challenged with rather high concentrations (10^−5^M) of synthetic non-sulphated Cragi-CCKs. No signal was obtained with cells transfected with an empty vector or with a Gα16 expressing vector. Activation of Cragi-CCKRs with Cragi-CCKs appeared slightly more efficient in absence of Gα16 (Supplementary Fig. 2). Thus a series of synthetic sulphated (s) or non-sulphated (ns) forms of Cragi-CCKs were tested as potential ligands for Cragi-CCKRs. Non-sulphated Cragi-CCK1 activates Cragi-CCKR1 and Cragi-CCKR2 with half maximal effective concentrations (EC_50_) of 0.19.10^−6^M and 0.46.10^−6^M respectively. The mono-sulphated form [Y^8^S] Cragi-CCK1 was, with respect to Cragi-CCKR1 and Cragi-CCKR2, 90 and 50 times more potent than its non-sulphated counterpart (Fig. 4, Table 1). Interestingly, the di-sulphated form [Y^6^S-Y^8^S] Cragi-CCK1was even more potent (2 and 5 folds respectively). The synthetic mono-sulphated form [Y^6^S] Cragi-CCK1 was less potent on Cragi-CCKR1 and inactive on Cragi-CCKR2 at doses as high as 10^−5^M (Table 1). Furthermore, the sulphated form [Y^3^S] Cragi-CCK2 activated only Cragi-CCKR1 though at relatively high concentrations (EC_50 = _5 10^−5^M). No response was observed with high concentrations of the oyster GALRF-amide unrelated neuropeptide used as negative control^14^ (Fig. 4). A possible transduction via Gαs was investigated using a cAMP luminescence assay but none of the synthetic peptides activated the cAMP signalling pathway even at concentrations as high as 10^−5^M.

**Table 1:** Cragi-CCK respective EC_50_ for receptor activation.

Peptide Name	Cragi-CCKR1	Cragi-CCKR2
Cragi-CCK1	19.5 ± 2.0 10^−8^M	46.2 ± 6.7 10^−8^M
[Y^6^S] Cragi-CCK1	1.64 ± 0.01 10^−8^M	No activation
[Y^8^S] Cragi-CCK1	0.229 ± 0.155 10^−8^M	1 ± 0.19 10^−8^M
[Y^6^S-Y^8^S] Cragi-CCK1	0.11 ± 0.025 10^−8^M	0.198 ± 0.025 10^−8^M
Cragi-CCK2	>10^−4^M	≫10^−4^M
[Y^3^S] Cragi-CCK2	>5 10^−5^M	≫10^−4^M

### Gene expression of Cragi-CCK signalling components

**Figure 5:**
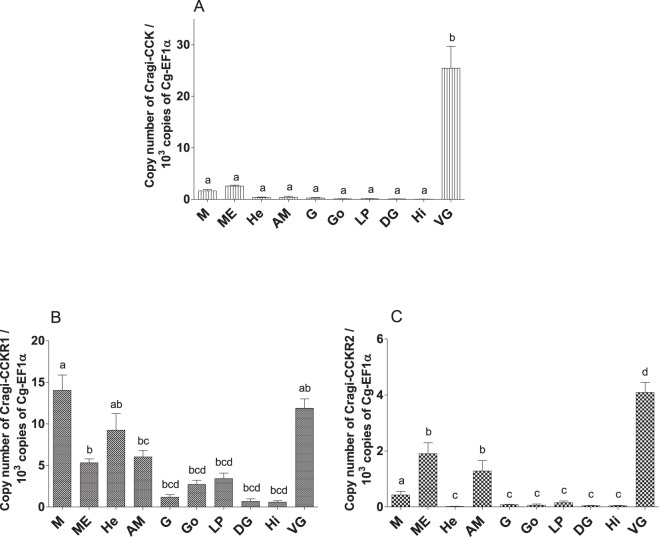
Distribution of mRNAs encoding Cragi-CCK (**A**), Cragi-CCKR1 (**B**) and Cragi-CCKR2 (**C**) in adult tissues. Each value is the mean + SEM of 5 pools of 6 animals. Expression levels were calculated as the number of copies of each specific transcript per 10^3^ copies of Elongation Factor 1 α (EF1α) mRNA. Results were statistically tested with a one-way ANOVA p < 0,05. Significantly different means are indicated by different letters. M: Mantle; ME: Mantle Edge; He: heart; AM: Adductor Muscle; G: Gills; Go: Gonad; LP: Labial Palps; DG: Digestive Gland; Hi: Hindgut; VG: Visceral Ganglia.

**Figure 6:**
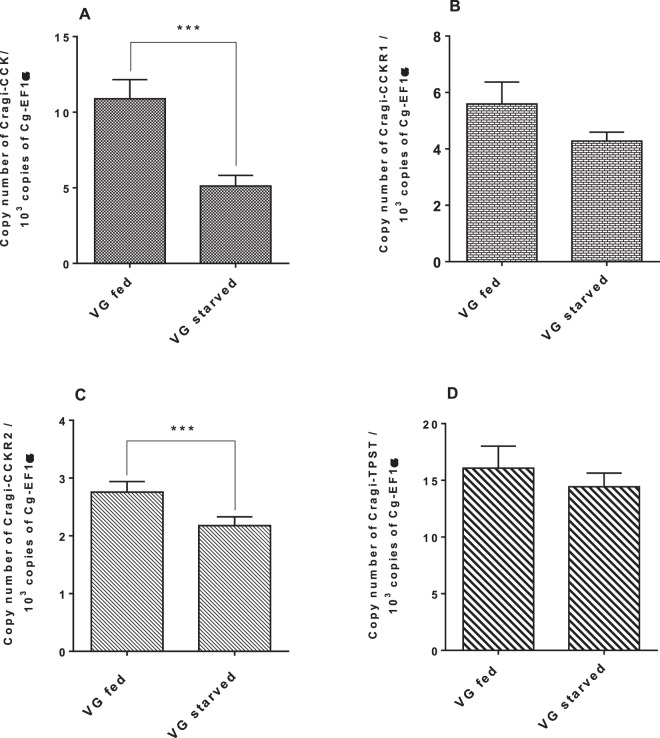
Expression levels of Cragi-CCK (**A**), Cragi-CCKR1 (**B**), Cragi-CCKR2 (**C**), Cragi-TPST (**D**) mRNAs in VG of four weeks *Isochrysis galbana* fed or starved oysters. Each value is the mean + SEM of 15 independent animals (VG after conditioning with or without food). Results were statistically tested with a student’s t test. Significantly different means of samples from fed and starved animals are indicated by *(p < 0,05) (**C**) and ***(p < 0.001) (**A**). No significant statistical difference was observed for (**B**,**D**).

**Figure 7:**
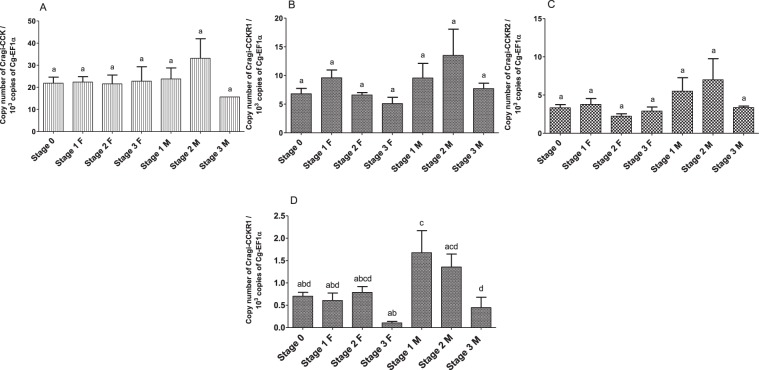
Level of expression of Cragi-CCK/Cragi-CCKR1/Cragi-CCKR2 mRNAs in VG (**A**–**C**) and of Cragi-CCKR1 mRNA in the gonads (**D**) along an annual reproductive cycle. Each value is the mean + SEM of 5 pools of 6 animals. Results were statistically tested with a one-way ANOVA, p < 0,05. Samples with significant statistical difference are marked with distinct letters. F: Female; M: Male; 0: stage 0 (sexual resting stage); 1: stage 1 (gonial multiplication stage); 2: stage 2 (tubule development and maturation stage); 3: stage 3 (sexual maturity stage).

The expression of Cragi-CCKR1, Cragi-CCKR2 and Cragi-CCK genes was investigated by RT-qPCR in several adult tissues and in fed and starved animals. Cragi-CCK gene was almost exclusively expressed in the visceral ganglia and to a lesser extent in the mantle (Fig. 5A). Cragi-CCKR1 gene was ubiquitously expressed in adult tissues though with a higher expression in the heart, the mantle and the visceral ganglia (Fig. 5B). In comparison, Cragi-CCKR2 was overall expressed at a lower level in adult tissues though at higher levels in the visceral ganglia, the mantle and the adductor muscle (Fig. 5C). To investigate the possible involvement of the oyster CCK signalling system in feeding regulation, we assessed the expression of the genes encoding the CCK signalling components including the major enzyme implicated in protein sulfation (TPST: Tyrosyl Protein sulfotransferase) in fed and four weeks starved animals. Both Cragi-CCK and Cragi-CCKR2 genes were significantly more expressed in VG of fed animals than in starved animals. Expression of all the other tested genes also slightly declined in starved animals yet not significantly (Fig. 6). Outside the VG, Cragi-CCKR1 decreased significantly in the gonad of starved animals (Supplementary Table 4). Given the expression of Cragi-CCKR1 in the gonad, we investigated the expression of receptor and ligand transcripts along a reproductive cycle. Expression of Cragi-CCK and Cragi-CCKRs did not fluctuate significantly along a reproductive cycle in VG. In contrast Cragi-CCKR1 gene showed a higher expression in the gonad during the first stages of reproduction and a significant decline of expression at the maturity stage (stage 3) in both males and females (Fig. 7).

### Myotropic activity assays

**Figure 8:**
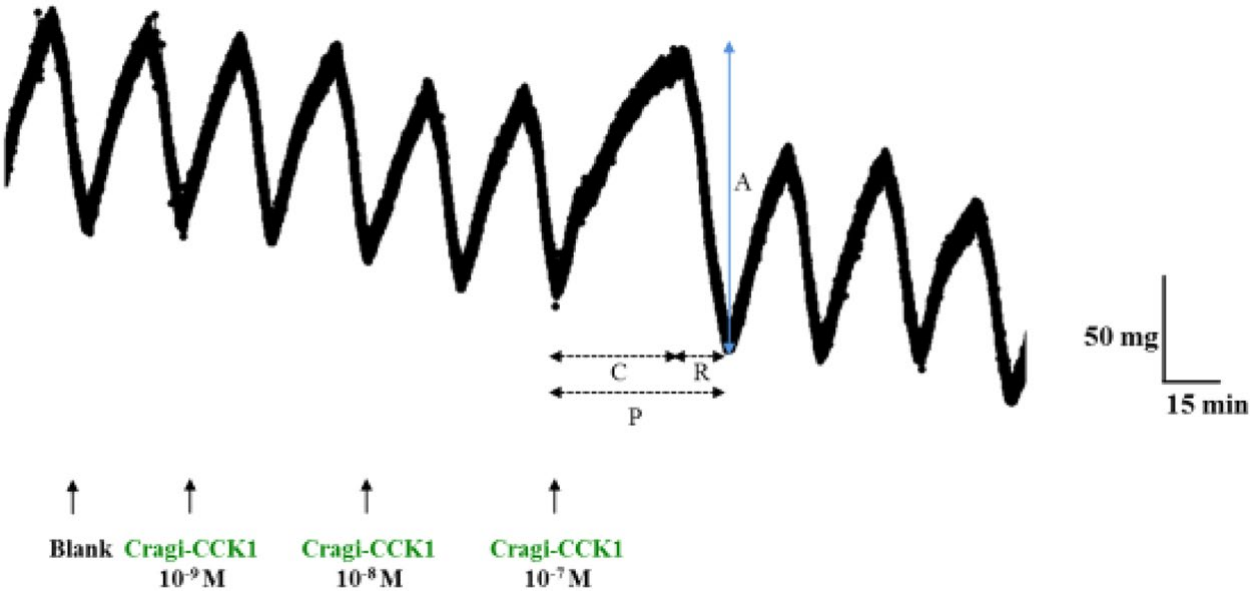
(**A**) Representative record of the hindgut-stimulating activity of Cragi-CCK1. Arrows indicate the application of saline solution (Blank) or Cragi-CCK1 peptide fractions at different concentrations. The different parameters taken into account to measure the biological response are indicated (C: Contraction time, R: Relaxation time, P: Period (min.) A: Amplitude of contraction (mg).

Bioassays were carried out on the oyster hindgut. The basal activity is cyclic and composed of a succession of contraction and relaxation phases of an average period of 30 minutes (Fig. 8). Different concentrations of [Y^6^S] Cragi-CCK1, [Y^8^S] Cragi-CCK1 and [Y^6^S-Y^8^S] Cragi-CCK1 were administered. All peptides except [Y^6^S] Cragi-CCK1 induce organ contraction though with distinct threshold concentrations. A minimal threshold concentration of 10^−10^M was obtained for [Y^8^S] Cragi-CCK1, 10^−9^M for [Y^6^S-Y^8^S] Cragi-CCK1 and to 10^−7^M for Cragi-CCK1 (Table 2). No response was obtained with [Y^6^S] Cragi-CCK1 at a concentration up to 10^−5^M. Active peptides induce a significant increase of the contraction and relaxation duration (except for [Y^8^S] Cragi-CCK1) and a significant increase of amplitude of contraction when a threshold concentration of [Y^6^S-Y^8^S] Cragi-CCK1 is administered. No effect was observed with FMRF-amide at a concentration of 10^−7^M.

**Table 2:** Effect of Cragi-CCK peptide variants on hindgut contraction parameters.

Peptide Name	Treatment	Duration (min)						Amplitude (A) (g)	
		Period (P)		Contraction (C)		Relaxation (R)			
Cragi-CCK1	No peptide	30,67 ± 0,67	***	20,18 ± 0,33	***	10,48 ± 0,78	*	0,161 ± 0,038	ns
	Peptide 10^−7^M	51,27 ± 0,15		35,23 ± 1,32		16,03 ± 1,13		0,203 ± 0,038	
[Y^6^S] Cragi-CCK1	No peptide	31,53 ± 1,45	ns	20,18 ± 0,98	ns	11,35 ± 0,85	ns	0,129 ± 0,019	ns
	Peptide 10^−5^M	32,23 ± 1,53		21 ± 1,42		11,23 ± 0,60		0,104 ± 0,021	
[Y^8^S] Cragi-CCK1	No peptide	29,66 ± 2,22	***	18,58 ± 1,03	***	11,08 ± 2,27	ns	0,121 ± 0,025	ns
	Peptide 10^−10^M	54,75 ± 1,97		37,13 ± 3,28		17,62 ± 5,27		0,173 ± 0,023	
[Y^6^S-Y^8^S] Cragi-CCK1	No peptide	30,57 ± 2,22	***	18,76 ± 1,07	*	11,80 ± 1,63	***	0,103 ± 0,006	*
	Peptide 10^−9^M	62,39 ± 2,50		38,57 ± 3,53		23,82 ± 1,35		0,153 ± 0,016	

Each value is the mean ± SEM of four experiments. Results were statistically tested with a student’s t test, *p < 0,05, ***p < 0,001, ns: not significant.

## Discussion

Structural and functional homology between peptide members of both the chordate G/CCK and arthropod SK groups, together with the phylogenetic proximity of their cognate receptors, support a common origin of these two signalling systems in the bilaterian ancestor. The present report reveals for the first time the existence of a functional CCK/SK signalling system in Lophotrochozoa, one of the two major clades of protostome animals.

The occurrence of a lophotrochozoan CCK/SK signalling system was first investigated by mining the transcriptomic and genomic resources of the oyster *C. gigas*. Then a wide-ranging phylogenetic analysis including a large number of functionally characterised chordate CCKRs and ecdysozoan SKRs was performed, and clearly identified Cragi-CCKR1 and Cragi-CCKR2 as orthologues of ecdysozoan SKRs and chordate CCKRs. Both oyster receptor genes harboured an intron at a position conserved among all chordate and arthropod receptor genes, further strengthening the argument that they evolved from a common bilaterian ancestor^1,34^. At least two CCKRs or SKRs are present in most animal lineages. The type-1 and type-2 CCKR groups are clearly separated within vertebrates. Our phylogenetic analysis revealed that the two receptor types present in some arthropods and Lophotrochozoa not only clustered separately from the vertebrate receptors but also grouped in a phylum-specific way, consistent with independent duplication events that occurred at least after Ecdysozoa and Lophotrochozoa diverged. The situation was similar for the two related CioRs of the urochordate *Ciona intestinalis*^41^. In *C. gigas*, the two Cragi-CCKRs clustered together and separately from putative CCK/SK receptors from other molluscs or from annelids. As Cragi-CCKR genes were positioned in separate genome contigs, the duplication event was probably not recent and likely occurred in an ancestor of the bivalve lineage.

In line with the receptor/ligand co-evolution theory, we inferred that putative oyster neuropeptides previously identified based on their slight sequence homology with CCK/SK peptides^7^ might represent the cognate ligands for Cragi-CCKRs. These peptides, i.e. Cragi-CCK1 and Cragi-CCK2, are generated from a single precursor^7^ displaying an organization similar to their mollusc^8^, annelid^42^, nematode^43^ and arthropod counterparts^34^. But their precursors are distinct from the chordate G/CCK precursors which only contain one peptide that can be processed into a variety of N-terminally extended mature forms^34,38^. All chordate G/CCKs share the common C-terminal GWMDF-amide motif, which is not conserved in the other animal groups. For example, non-chordate deuterostomian putative CCK/SK-like peptides display a GXXF-amide motif^44^, the *C. elegans* homologue a QF-amide motif ^27^, although all arthropod SKs display the C-terminal core sequence YGHMRF-amide motif^34^. Thus, in addition to the basically conserved (D/E)Y(G) sequence, the F-amide terminal motif appears as the common feature shared by bilaterian CCK/SK family members^44^. Hence Cragi-CCK1 resembles SKs because it exhibits a C-terminal RF-amide. In contrast, Cragi-CCK2 represents a singular exception because it displays an atypical C-terminal RW-amide motif. A comparison of Cragi-CCKs with other family members revealed the presence of one or even two (for Cragi-CCK1) conserved tyrosine residues that are frequently post-translationally sulphated^22^. Since both tyrosine residues are located in a consensus sequence compatible for recognition as TPST substrates^45^, Cragi-CCKs were synthesized in their sulphated and non-sulphated forms. This report clearly demonstrates that the different sulphated forms of Cragi-CCK1 specifically activated both Cragi-CCKR1 and Cragi-CCKR2 and were far more potent than their non-sulphated counterparts. Interestingly, similarly to the cionin signalling system^41^, the disulphated form of Cragi-CCK1 was more potent than its monosulphated derivatives. The sulphate moiety also appeared critical for receptor activation in other CCK/SK systems^38,46,47^, although it was not found essential for the activation of *C. elegans* CKRs^27^ or mammalian gastrin/CCK2R^22^. Both Cragi-CCKRs possess a conserved arginine residue in the extra-cellular loop 2. In mammalian CCK1R, it participates in the interaction with the negatively charged sulphate moiety of CCK^48^. Interestingly, CCK2R does not share this arginine residue, and neither do *C. elegans* CKRs (Fig. 1). The biochemical characterisation of Cragi-CCKs presented in this study clearly proves the existence of both mono and di-sulphated forms in the VG, consistent with the expression of the TPST gene in this tissue. Mass spectrometry signals associated with the sulphated forms were rather weak as compared to those of the non-sulphated form, suggesting that the former were expressed in lower quantities. However, mass spectrometry was not quantitative, and moreover this situation may have resulted from partial desulphation due to the acidic conditions used in the initial extraction process and in source dissociation^45^. Our experimental conditions did not allow us to discriminate between [Y^6^S] Cragi-CCK1 and [Y^8^S] Cragi-CCK1. Both forms were likely to be present, resulting either from partial desulphation of the disulphated form or representing intermediary molecular species of the synthesis of the di-sulphated form. Of the two monosulphated forms, [Y^8^S] Cragi-CCK1 was more potent than [Y^6^S] Cragi-CCK1, suggesting that the sulphated moiety located on the tyrosine residue with the most conserved position could be an ancestral feature, as suggested for cionin^41^. Thus, the sulphated forms of Cragi-CCK likely represent physiologically active peptides in oyster, even though the non-sulphated Cragi-CCK1 form may also be biologically relevant given its potency to activate Cragi-CCKRs. Indeed, in the context of local release from nerve terminals, a rather high concentration of peptide can be expected *in vivo* at the vicinity of the receptors. This assumption is fully compatible with a role of Cragi-CCK1 as a neurotransmitter, as formerly demonstrated for CCK^22^ and SK^49^ peptides. Regulatory activity from non-sulphated SKs was also shown in insects^50,51^. All oyster peptides activated calcium accumulation in Cragi-CCKR-expressing cells, suggesting that these receptors couple to G proteins of the Gα_q/11_ familly. Similar findings were observed for chordate CCKRs^38,41^ and SK family receptors^27,46,47^, even though CCK1R^52^, *C. elegans* CKR^27^, and the red flour beetle SKR^47^ also activated the cAMP transduction pathway. Activation of other signaling pathways cannot be ruled out, e.g. the JAK/STAT-dependent pathway: the activation of this pathway for CCK2R requires Gα_q_ proteins and the NPXXY motif, which is also present in the Cragi-CCKR1 sequence^38^. Taken together, these results clearly show that the neuropeptide/receptor pairs Cragi-CCK1/Cragi-CCKR1 and Cragi-CCKR2 represent a Lophotrochozoan orthologue of the CCK/SK signalling systems of chordates and ecdysozoa.

In other respects, Cragi-CCK2 forms did not act as specific ligands for Cragi-CCKR1 and Cragi-CCKR2 since the concentrations required to elicit a partial activation of the receptors appear not physiologically relevant except conceivably in the specific context of synaptic transmission. This raises the issue of the nature of the cognate receptor(s) for this neuropeptide form which logically should display a relative phylogenetic vicinity to Cragi-CCKR1/2. The type of interaction between the two Cragi-CCK signalling systems is particularly intriguing since both peptides represent end products of the same precursor.

CCK/SK signalling systems regulate gut functioning, food ingestion, satiety, as well as a variety of other biological processes, so that these neuropeptides are now recognized as multifunctional regulators^22,53^. In oyster, the Cragi-CCK gene was predominantly expressed in nervous tissues, as expected for a neuropeptide-encoding gene. In contrast, Cragi-CCKRs were widely expressed in many tissues but with distinct patterns. Cragi-CCKR1 was expressed in almost all tissues though at variable levels, Cragi-CCKR2 was less expressed and mostly in the VG, in richly innervated tissues such as the adductor muscle and the mantle edge or in tissues containing neurons such as the mantle. These observations support a multifunctional activity for Cragi-CCKR1 signalling, and a more restricted role possibly associated with neuroregulation for Cragi-CCKR2 signalling.

Predominant expression of the Cragi-CCKR1 gene in oyster heart is reminiscent of the role of SKs in heart activity in insects^29^. In the same way, the expression of the Cragi-CCKR1 gene in oyster digestive tissues, albeit at moderate levels, is in agreement with the fact that SKs induce the release of α-amylase from the scallop digestive gland^37^ or with the recorded activity of both sulphated and non-sulphated Cragi-CCK1 peptides on oyster hindgut motility. Interestingly, Cragi-CCK1 peptides increase the period of spontaneous hindgut contractions *in vitro* but chiefly did not affect their amplitude, thus contrasting with the stimulatory effect of SKs on insect hindgut motility^54,55^, but in line with the *in vivo* inhibition of foregut or larval anterior midgut contraction by SKs in *Drosophila*^51^. Cragi-CCK1 peptides seem to decrease the emptying process of the intestine in oyster. Thus, it would be interesting to test whether the anterior parts of the gut also respond to these peptides since different responses were noticed in different insect species^51,56^. By negatively controlling hindgut activity, Cragi-CCK1s probably affect food intake in oyster. The increased expression of Cragi-CCK1 and Cragi-CCKR2 in fed oysters as compared to starved oysters also pleads for a possible role of the Cragi-CCK system in satiety signalling, as also revealed in *Drosophila*^57^ and other insects^31,33,58^. Besides the pleiotropic activities of CCK/SK peptides^22,53^, it was intriguing to find a differential expression of Cragi-CCKR1 in the gonad area along the reproductive cycle and according to the nutritional status. Cragi-CCKR1 expression declined as gametogenesis progressed. Therefore, this receptor probably plays a role in glycogen storage cells since this tissue almost completely disappears in the mature gonad. SK signalling in Ecdysozoa is involved in the regulation of fat^27^ and energy storage^50^, and expression of Cragi-CCKR1 is higher in the gonad of fed oysters. Therefore it is tempting to propose a role for the Cragi-CCK signalling system as an additional regulator of the storage metabolism that might connect feeding and storage processes, as recently proposed for the sNPF-like signalling system in *C. gigas*^14^.

This study outlines the lophotrochozoan evolutionary version of the CCK/SK signalling system and underlines its remarkable conservation at the structural and functional levels, implying a central role of CCK/SK in physiological regulation in bilateria.

## Material and Methods

### Animals and tissue sampling

Two-year old adult oysters *C. gigas*, purchased from a local farm (Normandie, France), were used for peptide characterization and transcription analyses. Stages of reproduction (Stage 0: resting undifferentiated stage, Stage (1): gonial multiplication stage, Stage (2): maturation stage, Stage (3): sexual maturity) were determined by histological analysis of gonad sections as described previously^59^. To study the influence of trophic conditions, one-year-old adult oysters were reared in water tanks either in absence of food or in presence of *Isochrysis galbana* (clone T-Iso) maintained at a concentration of 6 million of cells/mL during (4) weeks. Adult tissues (mantle, gills, labial palps, digestive gland, hindgut, gonad (mix of all stages), heart, adductor muscle) were sampled, the visceral ganglia (VG) were carefully dissected out, thus limiting any contamination from the adjacent adductor muscles. All the samples were either placed in TriReagent (Sigma) or stored at −80 °C until use. For expression studies, adult tissues or VG and gonads during gametogenesis from (6) animals were mixed to generate (5) pools of each tissue. Individual VG from 15 fed or starved animals were used to study gene expression.

### Peptide synthesis

All peptides were custom-synthesized by GeneCust (Luxemburg) using a standard Fmoc solid-phase protocol (Supplementary Table 1). Sulphated peptide variants were synthesized by coupling Fmoc-Tyr(SO_3_Na)-OH amino acids instead of Fmoc-tyr(tBu)-OH amino acids. The sequences of *C. gigas* peptides were obtained from an in-house peptide database yielded by MS analyses of tissue extracts and data mining^7^.

### *In silico* analyses

Multiple sequence alignment was performed with CCKRs from various species (Supplementary Table 2) using MUSCLE^60^. The alignment was manually trimmed that spans from the first to the seventh transmembrane domains. To determine the relationship between Cragi-CCKRs and CCKRs from other species, phylogenetic and molecular evolutionary analyses were conducted using MEGA version 6^61^ based on the maximum likelihood method. The reliability of the inferred trees was estimated by applying the bootstrap procedure with 1000 replications.

### Reverse endocrinology

#### Molecular cloning of the Cragi-CCKRs and transfection of mammalian cells

BLAST analysis of *C. gigas* transcriptomic database “GigaTon”^11^ using *Drosophila* SKR as query resulted in the identification of two full length cDNAs encoding Cragi-CCKR1 and Cragi-CCKR2 (CHOYP_CCKAR.3.6 and CHOYP_CCKAR.4.6) respectively. The CDS of the Cragi-CCKR genes were amplified by PCR (Pfu DNA polymerase, Promega) using gene-specific sense primers harbouring a Kozak consensus sequence and antisense primers (Supplementary Table 3). Ten nanogram of plasmid DNA (Pal 17.3 vector, Evrogen) from a *C. gigas* “all developmental stages and adult central nervous system” directional and normalized cDNA library^62^ was used as template. The resulting PCR products were directionally cloned into the pcDNA3.1 expression vector (Invitrogen). The correct insertion of the PCR products was confirmed by sequencing. Human Embryonic Kidney (HEK293T) cells were transiently transfected with the Cragi-CCKR/pcDNA3.1 constructs using Fugene HD (Promega) according to the manufacturer’s instructions. As a first step, co-transfection was done with a pcDNA3.1 expression construct for the human Gα_16_ subunit, a promiscuous G protein that can direct intracellular signalling of GPCRs to the release of calcium via the phospholipase C_β_ pathway, regardless of the endogenous G protein coupling of the receptor^63^. To assess receptor activity independent of Gα_16_, calcium responses were measured in cells expressing only the Cragi-CCKRs. Cells for negative control experiments were transfected with empty pcDNA3.1 and Gα_16_/pcDNA3.1 constructs.

#### Calcium fluorescence assay

Activation of the Cragi-CCKRs by candidate peptide ligands was monitored using a fluorescence-based calcium mobilization assay. Briefly, transfected HEK293T cells were loaded with Fluo-4 Direct (Invitrogen) plus probenecid (qsp 2.5 mM final in the cell) (Molecular Probes) for 1 hour (45 min at 37 °C and 15 min at room temperature). Excitation of the fluorophore was done at 488 nm. The calcium response was measured for 2 min at 525 nm using a FLEXstation 3 (Molecular Devices) at 37 °C. Data were analysed using SoftMax Pro (Molecular Devices). Candidate ligands were first tested at a final concentration of 10^−5^ M. Concentration-response measurements of activating ligands were conducted in quadruplicate and for at least three independent experiments. Half maximal effective concentrations (EC_50_ values) were calculated from concentration-response curves that were constructed using nonlinear regression analysis with a sigmoidal dose-response equation using Prism 5.0 (GraphPad software, USA).

#### cAMP luminescence assay

Cragi-CCKR transfected HEK 293 T cells were incubated with Glosensor cAMP reagent (qsp 4% final in the medium) (Promega) for 2 hours at room temperature prior to the injection of the candidate ligands. cAMP luminescence response was measured for 30 min after injection using a FLEX station 3 (Molecular Devices) at room temperature. Data were analysed using SoftMax Pro (Molecular Devices). Candidate peptide ligands were first tested at a final concentration of 10^−5^ M.

### Reverse transcription and quantitative PCR

RT-qPCR analysis was performed using the iCycler iQ© apparatus (Bio-Rad). Total RNA was isolated from adult tissues using Tri-Reagent (Sigma-Aldrich) according to the manufacturer’s instructions. Recovered RNA was then further purified on Nucleospin RNAII columns (Macherey-Nagel). After treatment during 20 min at 37 °C with 1 U of DNase I (Sigma) to prevent genomic DNA contamination, 1 μg of total RNA was reverse transcribed using 1 μg of random hexanucleotidic primers (Promega), 0.5 mM dNTPs and 200 U MMuLV Reverse Transcriptase (Promega) at 37 °C for 1 h in the appropriate buffer. The reaction was stopped by incubation at 70 °C for 10 min. The GoTaq® qPCR Master Mix (Promega) was used for real time monitoring of amplification (5 ng of cDNA template, 40 cycles: 95 °C/15 s, 60 °C/15 s) with gene-specific primers (Supplementary Table 2). A parallel amplification of *C. gigas* Elongation Factor 1α (EF1 α) transcript (BAD15289) was carried out to normalize the expression data of the studied transcripts. EF1 α was found as a reliable normalization gene as no significant difference (p < 0.05) of Ct values was observed between the different samples compared. Coefficient of variation of EF1 α was less than 5%. Thus, the relative level of each gene expression was calculated for one copy of the EF1 α reference gene by using the following formula: *N* = 2^(Ct EF1α − Ct *Cg*-cDNA)^. The PCR amplification efficiency (E; E = 10^(−1/slope)^) for each primer pair was determined by linear regression analysis of a dilution series to ensure that E ranged from 1.98 to 2.02. The specificity of the primer pairs was confirmed by melting curve analysis at the end of each qPCR run.

### Myotropic bioassay

The myotropic bioassay was performed using oyster’s hindgut according to the protocol described earlier for the cuttlefish oviduct^64^. The muscle chamber was perfused at a flow rate of 0.67 mL.min^−1^. Increasing concentrations of synthetic peptides (Cragi-CCK1, [Y^8^S] Cragi-CCK1, [Y^6^S] Cragi-CCK1, [Y^6^S-Y^8^S] Cragi-CCK1) were injected in the perfusing flow using a three-way valve to avoid mechanical stress. The flow of the samples into the muscle chamber was traced by adding phenol red.

### Statistical analysis

Gene expression levels between different tissues and between samples at different reproduction stages were compared using one-way ANOVA followed by a Tukey post hoc test. Expression levels between fed and starved animals were compared using an unpaired Student’s t test. Significance was set at p < 0.05.

### Purification and mass spectrometry analysis of endogenous Cragi-CCK1 forms

One hundred visceral ganglia, frozen and crushed in liquid nitrogen, were extracted in methanol/water/acetic acid (90/10/1 v/v/v) 30 minutes at 4 °C. After centrifugation 20 minutes at 15 000 g at 4 °C, supernatant was concentrated on C18 Sep-Pak cartridges. HPLC analysis was performed with a VARIAN-9012 solvent delivery system coupled to a VARIAN-9050 wave-length UV-VIS detector set at 214 nm. The extract was eluted with a 55-min linear gradient of 0.75% ACN *per* min from 22 to 58% ACN with TFA 0.1% on a C18 nucleodur column (4 mm × 250 mm × 5 µm). Fractions 45–48 corresponding to the retention times of the synthetic forms of CCK1 were pooled and separated on the same column with a 10-min linear gradient of 0.2% ACN per min from 45 to 47% ACN containing 10 mM ammonium acetate pH 5.2. Synthetic Cragi-CCK1, [Y^6^S] Cragi-CCK1 and [Y^6^S-Y^8^S] Cragi-CCK1 were also separated in the same conditions. Fractions 15 to 19 were analyzed by mass spectrometry. Analysis was realized in a triple quadrupole mass spectrometer with an electrospray interface (LCMS 8030Plus; Shimadzu), with selected ion monitoring (SIM) mode. Ions selected for non-sulphated Cragi-CCK1 in positive mode were [M + H] + 1644.7, [M + 2 H]2 + 822.5 and 833.6, the [M + 2 H]2 + with sodium adduct. For sulphated peptides, negative mode was selected with respectively singly charged and doubled charged ions corresponding to 1722.4 and 860.4 for [Y^6^S] Cragi-CCK1 and 1802.5 and 900.5 for [Y^6^S-Y^8^S] Cragi-CCK1.

## Electronic supplementary material

Tables S1-4 Fig S1

## References

[1] Mirabeau O, Joly J (2013). Molecular evolution of peptidergic signaling systems in bilaterians. Proc Natl Acad Sci USA.

[2] Jékely G (2013). Global view of the evolution and diversity of metazoan neuropeptide signaling. Proc Natl Acad Sci USA.

[3] Cox KJ (1997). Cloning, characterization, and expression of a G-protein-coupled receptor from *Lymnaea stagnalis* and identification of a leucokinin-like peptide, PSFHSWSamide, as its endogenous ligand. J Neurosci.

[4] Tensen CP (1998). The lymnaea cardioexcitatory peptide (LyCEP) receptor: a G-protein-coupled receptor for a novel member of the RFamide neuropeptide family. J Neurosci.

[5] Moroz LL (2006). Neuronal transcriptome of *Aplysia*: neuronal compartments and circuitry. Cell.

[6] Conzelmann M, Williams EA, Krug K, Franz-wachtel M, Macek B (2013). The neuropeptide complement of the marine annelid *Platynereis dumerilii*. BMC Genomics.

[7] Stewart MJ (2014). Neuropeptides encoded by the genomes of the Akoya pearl oyster *Pinctata fucata* and Pacific oyster *Crassostrea gigas*: a bioinformatic and peptidomic survey. BMC Genomics.

[8] Veenstra JA (2010). Neurohormones and neuropeptides encoded by the genome of *Lottia gigantea*, with reference to other mollusks and insects. Gen. Comp. Endocrinol..

[9] Zatylny-Gaudin C (2016). Neuropeptidome of the cephalopod *Sepia officinalis*: identification, tissue mapping, and expression pattern of neuropeptides and neurohormones during egg laying. J. Proteome Res..

[10] Zhang GG (2012). The oyster genome reveals stress adaptation and complexity of shell formation. Nature.

[11] Riviere G (2015). GigaTON: An extensive publicly searchable database providing a new reference transcriptome in the pacific oyster *Crassostrea gigas*. BMC Bioinformatics.

[12] Bauknecht P, Jékely G (2015). Large-scale combinatorial deorphanization of *Platynereis* neuropeptide GPCRs. Cell Rep..

[13] Conzelmann M (2013). Conserved MIP receptor-ligand pair regulates *Platynereis* larval settlement. Proc Natl Acad Sci USA.

[14] Bigot L (2014). Functional characterization of a short neuropeptide F-related receptor in a lophotrochozoan, the mollusk *Crassostrea gigas*. J. Exp. Biol..

[15] Li, S. *et al*. Adipokinetic hormones and their G protein-coupled receptors emerged in Lophotrochozoa. *Scientific Reports* 1–13, 10.1038/srep32789 (2016). 10.1038/srep32789 PMC5024129 27628442

[16] Dubos M-P (2018). Characterization of a tachykinin signalling system in the bivalve mollusc *Crassostrea gig*as. Gen. Comp. Endocrinol..

[17] Johnsen AH (1998). Phylogeny of the Cholecystokinin/Gastrin Family. Front. Neuroendocrinol..

[18] Makhlouf GM, McManus JP, Card WI (1964). The action of gastrin II on gastric-acid secretion in man. Comparison of the maximal secretory response to gastrin II and histamine. Lancet (London, England).

[19] Koh TJ, Chen D (2000). Gastrin as a growth factor in the gastrointestinal tract. Regul. Pept..

[20] Schmidt WE (1991). Role of CCK in regulation of pancreaticobiliary functions and GI motility in humans: effects of loxiglumide. Am. J. Physiol..

[21] Gibbs J, Young RC, Smith GP (1973). Cholecystokinin elicits satiety in rats with open gastric fistulas. Nature.

[22] Rehfeld JF (2017). Cholecystokinin-From local gut hormone to ubiquitous messenger. Front. Endocrinol. (Lausanne)..

[23] Nachman RJ, Holman G, Haddon WF, Ling N (1986). Leucosulfakinin, a sulfated insect neuropeptide with homology to Gastrin and Cholecystokinin. Science (80-.)..

[24] Clynen E, Schoofs L (2009). Peptidomic survey of the locust neuroendocrine system. Insect Biochem. Mol. Biol..

[25] Zoephel J, Reiher W, Rexer K-H, Kahnt J, Wegener C (2012). Peptidomics of the agriculturally damaging larval stage of the cabbage root fly *Delia radicum* (Diptera: Anthomyiidae). PLoS One.

[26] Torfs P (2002). Isolation, identification, and synthesis of a disulfated sulfakinin from the central nervous system of an arthropods the white shrimp *Litopenaeus vannamei*. Biochem. Biophys. Res. Commun..

[27] Janssen T (2008). Discovery of a cholecystokinin-gastrin-like signaling system in nematodes. Endocrinology.

[28] Maestro JL (2001). Screening of antifeedant activity in brain extracts led to the identification of sulfakinin as a satiety promoter in the German cockroach. Are arthropod sulfakinins homologous to vertebrate gastrins-cholecystokinins?. Eur. J. Biochem..

[29] Nichols Ruthann (2009). Plasticity in the effects of sulfated and nonsulfated sulfakinin on heart contractions. Frontiers in Bioscience.

[30] Harshini S, Nachman RJ, Sreekumar S (2002). *In vitro* release of digestive enzymes by FMRF amide related neuropeptides and analogues in the lepidopteran insect *Opisina arenosella* (Walk.). Peptides.

[31] Zels S (2015). Sulfakinin is an important regulator of digestive processes in the migratory locust, *Locusta migratoria*. Insect Biochem. Mol. Biol..

[32] Wei Z (2000). Sulfakinins reduce food intake in the desert locust, *Schistocerca gregaria*. J. Insect Physiol..

[33] Meyering-Vos M, Müller A (2007). RNA interference suggests sulfakinins as satiety effectors in the cricket *Gryllus bimaculatus*. J. Insect Physiol..

[34] Yu N, Smagghe G (2014). CCK(-like) and receptors: Structure and phylogeny in a comparative perspective. Gen. Comp. Endocrinol..

[35] Vigna SR, Morgan JL, Thomas TM (1984). Localization and characterization of gastrin/cholecystokinin-like immunoreactivity in the central nervous system of *Aplysia californica*. J. Neurosci..

[36] Dhainaut-Courtois N, Dubois MP, Tramu G, Masson M (1985). Occurrence and coexistence in *Nereis diversicolor* O.F. Müller (Annelida Polychaeta) of substances immunologically related to vertebrate neuropeptides. Cell Tissue Res..

[37] NACHMAN RONALD J., GIARD WILFRID, FAVREL PASCAL, SURESH T., SREEKUMAR S., HOLMAN G. MARK (1997). Insect Myosuppressins and Sulfakinins Stimulate Release of the Digestive Enzyme ?-Amylase in Two Invertebrates: The Scallop Pecten maximus and Insect Rhynchophorus ferrugineus. Annals of the New York Academy of Sciences.

[38] Dufresne M, Seva C, Fourmy D (2006). Cholecystokinin and Gastrin Receptors. Physiol. Rev..

[39] Zatylny-Gaudin C, Favrel P (2014). Diversity of the RFamide peptide family in mollusks. Front. Endocrinol. (Lausanne)..

[40] Johnsen AH, Rehfeld JF (1990). Cionin: A disulfotyrosyl hybrid of cholecystokinin and gastrin from the neural ganglion of the protochordate *Ciona intestinalis*. J. Biol. Chem..

[41] Sekiguchi T, Ogasawara M, Satake H (2012). Molecular and functional characterization of cionin receptors in the ascidian, *Ciona intestinali*s: the evolutionary origin of the vertebrate cholecystokinin/gastrin family. J. Endocrinol..

[42] Veenstra JA (2011). Neuropeptide evolution: neurohormones and neuropeptides predicted from the genomes of *Capitella telet*a and *Helobdella robusta*. Gen. Comp. Endocrinol..

[43] McVeigh P, Leech S, Marks NJ, Geary TG, Maule AG (2006). Gene expression and pharmacology of nematode NLP-12 neuropeptides. Int. J. Parasitol..

[44] Elphick MR, Mirabeau O (2014). The Evolution and Variety of RFamide-TypeNeuropeptides: Insights from Deuterostomian Invertebrates. Front. Endocrinol. (Lausanne)..

[45] Griffiths, J. R. & Unwin, R. D. Analysis of protein post-translational modifications by mass spectrometry. (Wiley and Sons, 2016).

[46] Kubiak TM (2002). Cloning and functional expression of the first *Drosophila melanogaster* sulfakinin receptor DSK-R1. Biochem. Biophys. Res. Commun..

[47] Zels Sven, Verlinden Heleen, Dillen Senne, Vleugels Rut, Nachman Ronald J., Broeck Jozef Vanden (2014). Signaling Properties and Pharmacological Analysis of Two Sulfakinin Receptors from the Red Flour Beetle, Tribolium castaneum. PLoS ONE.

[48] Gigoux V (1999). Arginine 197 of the cholecystokinin-A receptor binding site interacts with the sulfate of the peptide agonist cholecystokinin. Protein Sci..

[49] Duve H (1995). The sulfakinins of the blowfly *Calliphora vomitoria*. Peptide isolation, gene cloning and expression studies. Eur. J. Biochem..

[50] Slocinska M, Marciniak P, Jarmuszkiewicz W, Rosinski G (2015). New metabolic activity of the nonsulfated sulfakinin Zopat-SK-1 in the insect fat body. Peptides.

[51] Nichols R (2007). The first nonsulfated sulfakinin activity reported suggests nsDSK acts in gut biology. Peptides.

[52] Yule DI, Tseng MJ, Williams JA, Logdson CD (1993). A cloned CCK-A receptor transduces multiple signals in response to full and partial agonists. Am. J. Physiol..

[53] Nässel DR, Williams MJ (2014). Cholecystokinin-like peptide (DSK) In *Drosophila*, not only for satiety signaling. Front. Endocrinol. (Lausanne)..

[54] Nachman RJ, Holman GM, Cook BJ, Haddon WF, Ling N (1986). Leucosulfakinin-II, a blocked sulfated insect neuropeptide with homology to cholecystokinin and gastrin. Biochem. Biophys. Res. Commun..

[55] Al-Alkawi H, Lange AB, Orchard I (2017). Cloning, localization, and physiological effects of sulfakinin in the kissing bug. Rhodnius prolixus. Peptides.

[56] Haselton AT, Yin C-M, Stoffolano JG (2006). The effects o*f Calliphora vomitoria* Tachykinin-I and the FMRFamide-related peptide Perisulfakinin on female *Phormia regina* crop contractions, *in vitro*. J. Insect Physiol..

[57] Söderberg JAE, Carlsson MA, Nässel DR (2012). Insulin-producing cells in the *Drosophila* brain also express satiety-inducing cholecystokinin-like peptide, drosulfakinin. Front. Endocrinol. (Lausanne)..

[58] Yu N, Nachman RJ, Smagghe G (2013). Characterization of sulfakinin and sulfakinin receptor and their roles in food intake in the red flour beetle *Tribolium castaneum*. Gen. Comp. Endocrinol..

[59] Rodet F, Lelong C, Dubos M-P, Costil K, Favrel P (2005). Molecular cloning of a molluscan gonadotropin-releasing hormone receptor orthologue specifically expressed in the gonad. Biochim Biophys Acta.

[60] Edgar RC (2004). MUSCLE: multiple sequence alignment with high accuracy and high throughput. Nucleic Acids Res..

[61] Tamura K, Stecher G, Peterson D, Filipski A, Kumar S (2013). MEGA6: Molecular evolutionary genetics analysis version 6.0. Mol. Biol. Evol..

[62] Fleury E (2009). Generation and analysis of a 29, 745 unique Expressed Sequence Tags from the Pacific oyster *(Crassostrea gigas)* assembled into a publicly accessible database: the GigasDatabase. BMC Genomics.

[63] Offermanns S, Simon MI (1995). G alpha 15 and G alpha 16 couple a wide variety of receptors to phospholipase C. J. Biol. Chem..

[64] Endress M (2018). Crustacean cardioactive peptides: Expression, localization, structure, and a possible involvement in regulation of egg-laying in the cuttlefish *Sepia officinalis*. Gen. Comp. Endocrinol..

[65] Thompson J, Higgins D, Gibson T (1994). CLUSTAL W: improving the sensitivity of progressive multiple sequence alignment through sequence weighting, position-specific gap penalties and weight matrix choice. Nucleic Acids Res..

