# Supplementary material for: Emergence of a cholecystokinin/sulfakinin signalling system in Lophotrochozoa
Source: Sci Rep. 2018 Nov 6;8:16424. doi: 10.1038/s41598-018-34700-4 (PMC6219549; doi:10.1038/s41598-018-34700-4)
Supplement: Supplementary file 1 — Tables S1-4 Fig S1 [file 41598_2018_34700_MOESM1_ESM.docx]

**Emergence of a cholecystokinin / sulfakinin signalling system in Lophotrochozoa**

Julie Schwartz^1^, Marie-Pierre Dubos^1^, Jérémy Pasquier^1^, Céline Zatylny-Gaudin^1^, and Pascal Favrel^1*^.

1. Normandie Université, UNICAEN, Sorbonne Universités, MNHN, UPMC, UA, CNRS 7208, IRD 207, Biologie des Organismes et Ecosystèmes Aquatiques (BOREA), CS14032, 14032 CAEN, Cedex 5, France.

**Supplementary table 1: Amino acid sequences of Cragi-CCK peptides.**

| Name | Amino acid sequence (NH_2_ to COOH) |
| --- | --- |
| Cragi-CCK1 | pEGAWDYDYGLGGGRFamide |
| [Y^8^S] Cragi-CCK1 | pEGAWDYDY(SO_3_H)GLGGGRFamide |
| [Y^6^S] Cragi-CCK1 | pEGAWDY(SO_3_H)DYGLGGGRFamide |
| [Y^6^S-Y^8^S] Cragi-CCK1 | pEGAWDY(SO_3_H)DY(SO_3_H)GLGGGRFamide |
| Cragi-CCK2 | FDYGGGRWamide |
| [Y^3^S] Cragi-CCK2 | FDY(SO_3_H)GGGRWamide |

**Supplementary table 2:** **Accession numbers and references of the CCKR sequences used for the alignment and the phylogenetic analysis**

(* indicates functionally characterized receptors)

| **Abbreviation** | **Species name** | **Accession number** | **Phylum** | **Reference** |
| --- | --- | --- | --- | --- |
| CCK1R | *Airluropoda melanoleuca* | XP_002924347.1 | Vertebrate | No reference |
| CCK2R | *Airluropoda melanoleuca* | XP_002925026.1 | Vertebrate | No reference |
| SKR | *Anopheles gambiae* | XP_001237203.1 | Ecdysozoa  (Arthropod) | S. Kobberup, C.J.P. Grimmelikhuijzen, Annotation for a putative Anopheles gambiae sulfakinin receptor., (Unpublished) |
| SKR | *Apis mellifera* | XP_006562432.1 | Ecdysozoa  (Arthropod) | No reference |
| SKR | *Bombyx mori* | NP_001127744.1 | Ecdysozoa  (Arthropod) | N. Yamanaka, S. Yamamoto, D. Zitnan, K. Watanabe, T. Kawada, H. Satake, Y. Kaneko, K. Hiruma, Y. Tanaka, T. Shinoda, H. Kataoka, Neuropeptide réception transcriptome reveals unidentified neuroendocrine pathways., PLoS ONE. 3 (2008). |
| CCK1R | *Bos taurus* | NP_001095335.1 | Vertebrate | A.V. Zimin, A.L. Delcher, L. Florea, D.R. Kelley, M.C. Schatz, D. Puiu, F. Hanrahan, G. Pertea, C.P. Van Tassell, T.S. Sonstegard, G. Marcais, M. Roberts, P. Subramanian, J.A. Yorke and S.L. Salzberg, A whole-genome assembly of the domestic cow, Bos Taurus., Genome Biol. 10 (2009). |
| CCK2R* | *Bos taurus* | NP_776687.2 | Vertebrate | M. Dufresne, C. Escrieut, P. Clerc, I. Le Huerou-Luron, H. Prats, V. Bertrand, V. Le Meuth, P. Guilloteau, N. Vaysse, D. Fourmy, Molecular cloning, developmental expression and pharmacological characterization of the CCKB/gastrin receptor in the calf pancreas., European journal of pharmacology. 297 (1996) 165-79. |
| CCKR | *Capitella capitata* | ELT89517.1 | Lophotrochozoa  (Annelida) | O. Simakoy, F. Marletaz, S.J. Cho, E. Edsinger-Gonzales, P. Havlak, U. Hellsten., D.H. Kuo, T. Larsson, J. Lv, D. Arendt, R. Savage, K. Osoegawa, P. de Jong, J. Grimwood, J.A. Chapman, H. Shapiro, A. Aerts, R.P. Otillar, A.Y. Terry, J.L. Boore, I.V. Grigoriev, D.R. Lindberg, E.C. Seaver, D.A. Weisblat, N.H. Putnam, D.S. Rokhsar, Insights into bilaterian evolution from three spiralian genomes. Nature. 493, (2013) 526-531. |
| CKR1 | *Caenorhabditis elegans* | NP_491918.3 | Ecdysozoa  (Nematoda) | T. Janssen, E. Meelkop, M. Lindemans, K. Verstraelen, S.J. Husson, L. Temmerman, R.J. Nachman, L. Schoofs, Discovery of a cholecystokinin-gastrin-like signaling system in nematodes., Endocrinology. 149 (2008) 2826-2839. |
| CKR2a* | *Caenorhabditis elegans* | ACA81683.1 | Ecdysozoa  (Nematoda) | T. Janssen, E. Meelkop, M. Lindemans, K. Verstraelen, S.J. Husson, L. Temmerman, R.J. Nachman, L. Schoofs, Discovery of a cholecystokinin-gastrin-like signaling system in nematodes., Endocrinology. 149 (2008) 2826-2839. |
| CKR2b* | *Caenorhabditis elegans* | ACA81684.1 | Ecdysozoa  (Nematoda) | T. Janssen, E. Meelkop, M. Lindemans, K. Verstraelen, S.J. Husson, L. Temmerman, R.J. Nachman, L. Schoofs, Discovery of a cholecystokinin-gastrin-like signaling system in nematodes., Endocrinology. 149 (2008) 2826-2839. |
| CCK1R | *Callithrix jacchus* | XP_002745977.1 | Vertebrate | No reference |
| CioR1* | *Ciona intestinalis* | NP_001027945.1 | Urochordate | T. Sekiguchi, M. Ogasawara, H. Satake, Molecular and functional characterization of cionin receptors in the ascidian, Ciona intestinalis: The evolutionary origin of the vertebrate cholecystokinin/gastrin family., Journal of endocrinology. 213 (2012) 99-106. |
| CioR2* | *Ciona intestinalis* | NP_001265909.1 | Urochordate | T. Sekiguchi, M. Ogasawara, H. Satake, Molecular and functional characterization of cionin receptors in the ascidian, Ciona intestinalis: The evolutionary origin of the vertebrate cholecystokinin/gastrin family., Journal of endocrinology. 213 (2012) 99-106. |
| Cragi-CCKR1* | *Crassostrea gigas* | MF787221 | Lophotrochozoa  (Mollusc) | Present publication |
| Cragi-CCKR2* | *Crassostrea gigas* | MF787222 | Lophotrochozoa  (Mollusc) | Present publication |
| CCK1R | *Danio rerio* | XP_697493.2 | Vertebrate | No reference |
| CCK2R | *Danio rerio* | CAQ14219.1 | Vertebrate | No reference |
| DSKR1* | *Drosophila melanogaster* | NP_001097021.1 | Ecdysozoa  (Arthropod) | T.M. Kubiak, M.J. Larsen, K.J. Burton, C.A. Bannow, R.A. Martin, M.R. Zantello, D.E. Lowery, Cloning and functional expression of the first Drosophila melanogaster sulfakinin receptor DSK-R1., Biochemical and biophysical research communications. 291 (2002) 313-320. |
| DSKR2* | *Drosophila melanogaster* | NP_001097023.1 | Ecdysozoa  (Arthropod) | R. Nichols. The first nonsulfated sulfakinin activity reported suggests nsDSK acts in gut biology., Peptides. 28 (2007) 767-773. |
| CCK1R | *Gallus gallus* | NP_001074970.1 | Vertebrate | [I.C. Dunn](https://www.ncbi.nlm.nih.gov/pubmed/?term=Dunn%20IC%5BAuthor%5D&cauthor=true&cauthor_uid=23443924), [S.L. Meddle](https://www.ncbi.nlm.nih.gov/pubmed/?term=Meddle%20SL%5BAuthor%5D&cauthor=true&cauthor_uid=23443924), [P.W. Wilson](https://www.ncbi.nlm.nih.gov/pubmed/?term=Wilson%20PW%5BAuthor%5D&cauthor=true&cauthor_uid=23443924), [C.A. Wardle](https://www.ncbi.nlm.nih.gov/pubmed/?term=Wardle%20CA%5BAuthor%5D&cauthor=true&cauthor_uid=23443924), [A.S. Law](https://www.ncbi.nlm.nih.gov/pubmed/?term=Law%20AS%5BAuthor%5D&cauthor=true&cauthor_uid=23443924), [V.R. Bishop](https://www.ncbi.nlm.nih.gov/pubmed/?term=Bishop%20VR%5BAuthor%5D&cauthor=true&cauthor_uid=23443924), [C.Hindar](https://www.ncbi.nlm.nih.gov/pubmed/?term=Hindar%20C%5BAuthor%5D&cauthor=true&cauthor_uid=23443924), [G.W. Robertson](https://www.ncbi.nlm.nih.gov/pubmed/?term=Robertson%20GW%5BAuthor%5D&cauthor=true&cauthor_uid=23443924), [D.W. Burt](https://www.ncbi.nlm.nih.gov/pubmed/?term=Burt%20DW%5BAuthor%5D&cauthor=true&cauthor_uid=23443924), [S.J.H. Ellison](https://www.ncbi.nlm.nih.gov/pubmed/?term=Ellison%20SJ%5BAuthor%5D&cauthor=true&cauthor_uid=23443924), [D.M. Morrice](https://www.ncbi.nlm.nih.gov/pubmed/?term=Morrice%20DM%5BAuthor%5D&cauthor=true&cauthor_uid=23443924), [P.M. Hocking](https://www.ncbi.nlm.nih.gov/pubmed/?term=Hocking%20PM%5BAuthor%5D&cauthor=true&cauthor_uid=23443924), Decreased expression of the satiety signal receptor CCKAR is responsible for increased growth and body weight during the domestication of chickens., AJP: Endocrinology and Metabolism. 304 (2013) E909-E921. |
| CCK2R* | *Gallus gallus* | NP_001001742.1 | Vertebrate | I.B.M. Nilsson, S.P.S. Svensson, H.J. Monstein, Molecular cloning of an unusual bicistronic cholecystokinin receptor mRNA expressed in chicken brain: A structural and functional expression study., Regulatory Peptides. 114 (2003) 37-43. |
| CCKR | *Helobdella robusta* | XP_009030452.1 | Lophotrochozoa  (Annelida) | O. Simakoy, F. Marletaz, S.J. Cho, E. Edsinger-Gonzales, P. Havlak, U. Hellsten., D.H. Kuo, T. Larsson, J. Lv, D. Arendt, R. Savage, K. Osoegawa, P. de Jong, J. Grimwood, J.A. Chapman, H. Shapiro, A. Aerts, R.P. Otillar, A.Y. Terry, J.L. Boore, I.V. Grigoriev, D.R. Lindberg, E.C. Seaver, D.A. Weisblat, N.H. Putnam, D.S. Rokhsar, Insights into bilaterian evolution from three spiralian genomes. Nature. 493, (2013) 526-531. |
| CCK1R* | *Homo sapiens* | NP_000721.1 | Vertebrate | C.D. Ulrich, I. Ferber, E. Holicky, E. Hadac, G. Buell, L.J. Miller, Molecular cloning and functional expression of the human gallbladder cholecystokinin A receptor., Biochemical and biophysical research communications. 191 (2013) 204-211. |
| CCK2R* | *Homo sapiens* | NP_795344.1 | Vertebrate | Y.M. Lee, M. Beinborn, E.W. McBride, M. Lu, L.F. Kolakowski,  A.S. Kopin, The human brain Cholecystokinin-B/gastrin receptor. Cloning and characterization. Journal of Biological Chemistry. 268 (1993) 8164-8169. |
| CCK1R | *Lottia gigantean* | XP_009059889.1 | Lophotrochozoa  (Mollusc) | O. Simakoy, F. Marletaz, S.J. Cho, E. Edsinger-Gonzales, P. Havlak, U. Hellsten., D.H. Kuo, T. Larsson, J. Lv, D. Arendt, R. Savage, K. Osoegawa, P. de Jong, J. Grimwood, J.A. Chapman, H. Shapiro, A. Aerts, R.P. Otillar, A.Y. Terry, J.L. Boore, I.V. Grigoriev, D.R. Lindberg, E.C. Seaver, D.A. Weisblat, N.H. Putnam, D.S. Rokhsar, Insights into bilaterian evolution from three spiralian genomes. Nature. 493, (2013) 526-531. |
| CCK2R | *Lottia gigantean* | XP_009047126.1 | Lophotrochozoa  (Mollusc) | O. Simakoy, F. Marletaz, S.J. Cho, E. Edsinger-Gonzales, P. Havlak, U. Hellsten., D.H. Kuo, T. Larsson, J. Lv, D. Arendt, R. Savage, K. Osoegawa, P. de Jong, J. Grimwood, J.A. Chapman, H. Shapiro, A. Aerts, R.P. Otillar, A.Y. Terry, J.L. Boore, I.V. Grigoriev, D.R. Lindberg, E.C. Seaver, D.A. Weisblat, N.H. Putnam, D.S. Rokhsar, Insights into bilaterian evolution from three spiralian genomes. Nature. 493, (2013) 526-531. |
| CCK3R | *Lottia gigantean* | XP_009047144.1 | Lophotrochozoa  (Mollusc) | O. Simakoy, F. Marletaz, S.J. Cho, E. Edsinger-Gonzales, P. Havlak, U. Hellsten., D.H. Kuo, T. Larsson, J. Lv, D. Arendt, R. Savage, K. Osoegawa, P. de Jong, J. Grimwood, J.A. Chapman, H. Shapiro, A. Aerts, R.P. Otillar, A.Y. Terry, J.L. Boore, I.V. Grigoriev, D.R. Lindberg, E.C. Seaver, D.A. Weisblat, N.H. Putnam, D.S. Rokhsar, Insights into bilaterian evolution from three spiralian genomes. Nature. 493, (2013) 526-531. |
| CCK1R | *Macaca mulatta* | XP_001084186.1 | Vertebrate | No reference |
| CCK2R | *Macaca mulatta* | XP_001102094.1 | Vertebrate | No reference |
| CCK1R* | *Mus musculus* | NP_033957.1 | Vertebrate | D. Ghanekar, E.M. Hadac,E.L. Holicky, L.J. Miller. Differences in partial agonist action at cholecystokinin receptors of mouse and rat are dependent on parameters extrinsic to receptor structure: molecular cloning, expression and functional characterization of the mouse type a cholecystokinin receptor.J Pharmacol Exp Ther.  282 (1997) 1206-1212. |
| CCK2R* | *Mus musculus* | NP_031653.1 | Vertebrate | L.C. Samuelson, M.S. Isakoff, K.A. Lacourse. Localization of the murine cholecystokinin A and B receptor genes. Mamm Genome. 6 (1995) 242–246. |
| CCK1R | *Pan troglodytes* | XP_526545.1 | Vertebrate | No reference |
| CCK2R | *Pan troglodytes* | XP_521813.1 | Vertebrate | No reference |
| CCK1R* | *Rattus norvegicus* | NP_036820.1 | Vertebrate | S.A. Wank, R. Harkins, R.T. Jensen, H. Shapira, A. de Weerth, T. Slattery, Purification, molecular cloning, and functional expression of the cholecystokinin receptor from rat pancreas., Proc Natl Acad Sci USA. 89 (1992) 3125-3129. |
| CCK2R* | *Rattus norvegicus* | NP_037297.1 | Vertebrate | J.R. Pisegna, A. De Weerth, K. Huppi, S.A. Wank, Molecular cloning of the human brain and gastric cholecystokinin receptor. Biochemical and biophysical research communications. 189 (1992) 296-303. |

**Supplementary table 3**: **Sequence of the primers used for cloning experiments and RT-qPCR.**

| Specific primer | Sequence (5’ to 3’) | Primer information |
| --- | --- | --- |
| s-*Cragi*-CCKR1 | 5’-**CACCATGA**ATAGTTCCAACATTTCCGAGTG-3’ | CDS fragment |
| as-*Cragi*-CCKR1 | 5’-TTAAACATGTTCTTGCGTCACTTCCGCGT-3’ | CDS fragment |
| s-*Cragi*-CCKR2 | 5’-**CACCATGG**CCTCCGAGAACCTG-3’ | CDS fragment |
| as-*Cragi*-CCKR2 | 5’-TTACTCAATGGACTGAACTCGTGTGTACTC-3’ | CDS fragment |
| Qs-*Cragi*-CCKR1 | 5′-GTATGCCCTTCAACATCATTCCA-3′ | qPCR |
| Qa-*Cragi*-CCKR1 | 5′-GTATCCGTTGGTGTCAGCAGT-3′ | qPCR |
| Qs-*Cragi*-CCKR2 | 5′-GGGAACGGGTTGGTGATT-3′ | qPCR |
| Qa-*Cragi*-CCKR2 | 5′-CCGTCCTCTGTATGCCCTTC-3′ | qPCR |
| Qs-*Cragi*-CCKs | 5′-CAGACACCAGAAAACAGCGTG-3′ | qPCR |
| Qa-*Cragi*-CCKs | 5′-GTCCCTCCAGCATAACGACAA-3′ | qPCR |
| Qs-*Cragi*-TPST | 5’-ACGGAAAGCCTGACCCAAA-3’ | qPCR Tyrosylprotein sulfotransferase |
| Qa-*Cragi*-TPST | 5’-GGCCAAAATCTCCTGCTCCT-3’ | qPCR Tyrosylprotein sulfotransferase |
| Qs-*Cg*-EF | 5′-ACCACCCTGGTGAGATCAAG-3′ | qPCR Elongation Factor 1 α |
| Qa*-Cg*-EF | 5′-ACGACGATCGCATTTCTCTT-3′ | qPCR Elongation Factor 1 α |

CDS: Coding DNA Sequences. s: sense primer. as: antisense primer**.** Nucleotides of the Kozak consensus sequence are in bold.

Table 4: **Expression levels of Cragi-CCKRs, Cragi-CCK, Cragi-TPST in tissues of four weeks fed and starved oysters**

|  | **Cragi-CCKR1** | | **Cragi-CCKR2** | | **Cragi-CCKs** | | **Cragi-TPST** | |
| --- | --- | --- | --- | --- | --- | --- | --- | --- |
|  | **Fed** | **Starved** | **Fed** | **Starved** | **Fed** | **Starved** | **Fed** | **Starved** |
| **Visceral ganglia** | 5,61 ± 1,52 | 4,35 ± 0,71 | 4,08 ± 1,54 | 2,04 ± 0,20 | 12,25 ± 2,06 | 6,10 ± 1,22 | 15,41± 1,83 | 22,66 ± 6,87 |
| **Mantle edge** | 3,12 ± 0,65 | 2,14 ± 0,31 | 1,86 ± 0,54 | 0,95 ± 0,20 | 2,04 ± 0,66 | 0,99 ± 0,34 | 12,84 ± 6,65 | 5,05 ± 0,96 |
| **Labial palps** | 1,82 ± 0,38 | 1,04 ± 0,23 | 0,28 ± 0,09 | 0,21± 0,07 | 0,13 ± 0,06 | 0.07 ± 0,03 | 3,96 ± 0,68 | 2,56 ± 0,44 |
| **Digestive gland** | 0,40 ± 0,09 | 0,24 ± 0,08 | 0,026 ± ,01 | 0,03 ± 0,01 | 0,06 ± 0,02 | 0,04 ± 0,01 | 3,64 ± 0,51 | 3,02 ± 0,2 |
| **Hindgut** | 0,78 ± 0,32 | 0,33± 0,15 | 0,04 ± 0,01 | 0,06 ± 0,02 | 0,03 ± 0,007 | 0,06 ± 0,02 | 1,33 ± 0,60 | 4,16 ± 1,75 |
| **Gonad** (Stages 1/2) | 1,42 ± 0,22 | 0,76 ± 0,11 | 0,07 ± 0,03 | 0,05 ± 0,01 | 0,14 ± 0,03 | 0,10 ± 0,04 | 5,82 ± 1,52 | 4,34 ± 0,31 |

Values are means ± SEM of the number of copies of a specific transcript per 10^3^ copies of elongation factor 1α (EF1α) mRNA. Grey-coloured shadings represent significantly different values (p<0.05, Student’s t test).

**Supplementary Figure 1:** Fluorescent signal induced by Cragi-CCKR1 and Cragi-CCKR2 expressed in HEK293T cells and challenged by Cragi-CCK1 at the concentration of 10^-5^M in absence (Cragi-CCKR1 / Cragi-CCKR2) or presence (Cragi-CCKR1 / Cragi-CCKR2+ G_α16_) of the promiscuous protein G_α16_. G_α16_ expressed alone or cells transfected with an empty vector were used as negative controls. Vertical bars represent the standard error of the mean (SEM), number of replicates n=3.
